# Molecular Identification of *Fusarium* Species in *Gibberella fujikuroi* Species Complex from Rice, Sugarcane and Maize from Peninsular Malaysia

**DOI:** 10.3390/ijms12106722

**Published:** 2011-10-11

**Authors:** Heng Mei Hsuan, Baharuddin Salleh, Latiffah Zakaria

**Affiliations:** 1 School of Biological Sciences, Universiti Sains Malaysia, 11800 USM, Pulau Pinang, Malaysia; E-Mails: hmsuan@yahoo.com (H.M.H.); sallehb@usm.my (B.S.); 2 Centre of Marine and Coastal Studies, Universiti Sains Malaysia (CEMACS-Mukahead), 11800 USM, Pulau Pinang, Malaysia

**Keywords:** *Fusarium*, rice, sugarcane, maize

## Abstract

The objective of this study was to identify *Fusarium* species in the *Gibberella fujikuroi* species complex from rice, sugarcane and maize as most of the *Fusarium* species in the species complex are found on the three crops. Isolates used were collected from the field and obtained from culture collection. The *Fusarium* isolates were initially sorted based on morphology and identifications confirmed based on the DNA sequence of the translation elongation factor 1-α (TEF-1α) gene. Based on the closest match of BLAST analysis, five species were recovered, namely, *F. sacchari*, *F. fujikuroi*, *F. proliferatum*, *F. andiyazi* and *F. verticillioides*. This is the first report regarding *F. andiyazi* from rice in Malaysia and Southeast Asia. The phylogenetic tree generated by using the neighbor joining method showed that isolates from the same species were grouped in the same clade. The present study indicated that *Fusarium* species in the *G. fujikuroi* species complex are widespread in rice, sugarcane and maize in Peninsular Malaysia. The findings also suggest that the use of morphological characters for identification of *Fusarium* species in the *G. fujikuroi* species complex from the three crops will lead to incorrect species designation.

## 1. Introduction

Diseases caused by *Fusarium* species in the *Gibberella fujikuroi* species complex are among the most common diseases reported on agricultural crops worldwide. In Peninsular Malaysia, several species of *Fusarium* in the species complex such as *F. verticillioides*, *F. sacchari*, *F. subglutinans*, *F. proliferatum* and *F. fujikuroi* have been implicated in diseases of several agricultural crops including rice, sugarcane and maize [1–3].

Studies of *Fusarium* species on agricultural crops in Peninsular Malaysia has been based mainly on morphological characters which could lead to incorrect species designation. There are limits on the use of morphological characters for the identification of species in the *G. fujikuroi* species complex as some species have very similar morphology, for example *F. proliferatum* and *F. fujikuroi*, *F. sacchari* and *F. subglutinans* as well as *F. verticillioides* and *F. andiyazi*. In spite of the limitations associated with morphological characters, these traits still play an important role in sorting isolates into smaller groups before other methods of identification are applied [4].

Differences in DNA sequences of genes have been used to support morphological identification of *Fusarium* species. Phylogenetic analysis on DNA sequences has been used to distinguish and to evaluate the genetic relationship among closely related *Fusarium* species [5–11].

The DNA sequences encoding translation elongation factor 1-α gene (TEF-1α) has been widely used for species identification. Sequences for this gene are available through GenBank and through the more focused FUSARIUM-ID [12]. The FUSARIUM-ID database contains sequences that can be associated with a strain that is publicly available through either USDA NRRL collection or the collection maintained at the Pennsylvania State University.

The correct species name of a plant pathogenic fungus is important for the development of effective disease control management, quarantine purposes and as a basis for making decisions to protect agricultural crops as well as other natural resources from fungal pathogens [13]. Therefore, the present study was conducted to identify *Fusarium* species in *G. fujikuroi* species complex isolated from rice, sugarcane and maize, by using morphological characteristics and DNA sequencing of TEF-1α gene. Based on similar studies conducted, we expected that diverse species of *G. fujikuroi* species complex would be identified.

## 2. Results and Discussion

The isolates in the stock cultures were identified solely based on morphological characteristics, which could easily result in misidentification. Based on the morphological characteristics of isolates from the field and reidentification of isolates from the stock culture, 30 isolates were identified as *F. verticillioides*, 20 as *F. sacchari*, seven as *F. subglutinans*, 12 as *F. proliferatum* and nine as *F. fujikuroi*. The *Fusarium* isolates showed white or purple cottony aerial mycelium. Various pigmentations from white to light purple and from purple to dark purple were observed. Most of the macroconidia were slender with three septate with a curved apical cell and a notched basal cell. The morphology of microconidia from all the isolates were oval shaped with flattened base and were mostly formed in false head. The length of the chain varied from short (less than 10 microconidia), to medium (10–20 microconidia) and long (more than 20 microconidia).

Although isolates of *Fusarium* species in *G. fujikuroi* species complex were difficult to distinguish, there are some morphological characteristics which could be used to differentiate these species. Isolates of *F. proliferatum* produced abundant aerial mycelia, pyriform and clavate conidia, polyphialides were frequent and the isolates were recovered from rice, sugarcane and maize. Isolates of *F. fujikuroi* produced cottony aerial mycelia, abundance of sporodochia, lesser polyphialides and only recovered from rice. The length of microconidia in chain of *F. proliferatum* was longer (more than 20) than *F. fujikuroi* (less than 20).

Isolates of *F. sacchari* and *F. subglutinans* were distinguished by their macroconidia. Isolates of *F. sacchari* isolated from rice and sugarcane produced pyriform macroconidia while isolates of *F. subglutinans* isolated from maize produced oval macroconidia. *F. sacchari* and *F. subglutinans* were differentiated from *F. verticillioides*, *F. fujikuroi* and *F. proliferatum* by the absence of microconidia in chain. Microconidia formation of *F. subglutinans* and *F. sacchari* were produced in false heads.

Problems with using morphological characteristics to identify *Fusarium* species in the *G. fujikuroi* species complex have been reported by many workers [14–17]. However, initial identification and characterization using morphological characters is important to sort the species into smaller groups before applying other methods of identification [4].

From PCR amplification with the EF-1 and EF-2 primers, a single band of 750 bp was successfully amplified from all 78 isolates of *Fusarium* spp. BLAST search for similarities using FUSARIUM-ID showed that the percentage of similarity of the isolates ranged from 97%–100%. Therefore, the species name assigned was according to the closest BLAST search (Table 1). Altogether, based on TEF-1α sequences, the closest match for all the isolates, 23 isolates were tentatively identified as *F. verticillioides*, 6 as *F. andiyazi*, 29 as *F. sacchari*, 11 as *F. fujikuroi* and 9 as *F. proliferatum* (Table 1).

Exact match or 100% similarity with isolates in FUSARIUM-ID database was found for only 12 isolates. Possible explanations were suggested by Geiser *et al*. [12] who suggested that the reason for this could be the occurrence of allelic variant, existence of a new species, no sequence representative in the database or that the query sequence is poorly defined.

Six isolates (3055, 3061, 3073, 3086, 3088 and 3137) from rice were tentatively identified as *F. andiyazi* as the TEF-1α sequences were similar to *F. andiyazi* with similarity ranging from 98%–99%. This report is the first of *F. andiyazi* occurrence in Malaysia as well as in Southeast Asia, and the first report of *F. andiyazi* on rice in Malaysia. *F. andiyazi* was first described by Marasas *et al.* [16] from sorghum in Africa and the United States and has also been reported from rice seed samples from Africa and Asia [10]. In the present study, swollen cells were observed, however, the swollen cells produced by *F. verticillioides* and pseudochlamydospores formed by *F. andiyazi* were difficult to distinguish which may explain why the structures were not easily distinguishable on CLA. *F. andiyazi* was also reported to be morphologically similar with *F. thapsinum*, a species in *G. fujikuroi* complex which is commonly found in sorghum [18]. Thus, *F. andiyazi* was very difficult to identify with only morphological characters and can only be distinguished by using DNA sequences. Wulff *et al*. [10] also differentiated *F. andiyazi* and *F. verticillioides* isolated from rice using TEF-1α sequences and both species were grouped in different clades. Another method to differentiate between *F. andiyazi* and *F. verticillioides* is by mycotoxins production. In a study by Wulff *et al*. [10], *F. verticillioides* produced significant level of fumonisin B1 whereas *F. andiyazi* only produced traces of fumonisions.

*F. verticillioides* is widely distributed worldwide and is one of the most common pathogens of maize, associated with ear rot and stalk rot of maize as well as symptomless infections or as endophyte on maize [19]. In the present study, only four isolates of *F. verticillioides* were isolated from rice infected with bakanae disease. In addition to agricultural crops, *F. verticillioides* has been isolated from mangrove soil in Malaysia [20]. In the present study the isolates of *F. verticillioides* were most commonly isolated from maize.

Isolates identified as *F. subglutinans* from maize based on morphological characteristics were all identified as *F. sacchari* based on TEF-1α sequences. Most of the isolates of *F. sacchari* in this study were isolated from sugarcane showing pokkah boeng disease symptoms although nine isolates were obtained from rice and two isolates from maize. *F. sacchari* causes pokkah boeng of sugarcane and is widely distributed in sugarcane growing areas in Malaysia [3]. Isolates of *F. sacchari* have also been isolated from rice with bakanae disease symptoms [2]. According to Burgess *et al*. [21], *F. subglutinans* is seldom isolated in warmer countries as it was widespread in cooler temperate regions. Logrieco *et al*. [22] also reported that *F. subglutinans* occurs under colder and more humid conditions. *F. subglutinans* and *F. sacchari* are distinct biological species [23] and it is possible that most, if not all of the reports of *F. subglutinans* from Malaysia are actually reports of *F. sacchari*.

**Figure 1:**
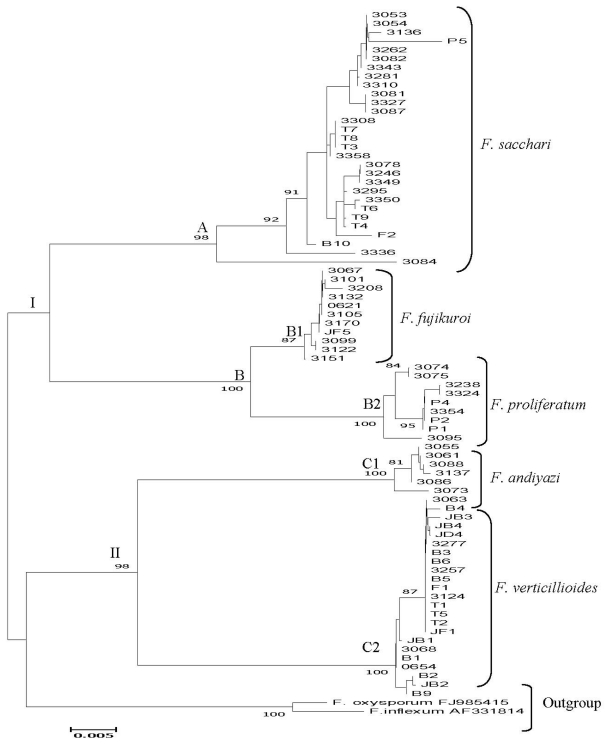
Neighbor-joining tree showing the relationship of 78 isolates of *Fusarium* of the *G. fujikuroi* species complex inferred from TEF-1α using Jukes-Cantor method. The bootstrap values (1000 replicates) higher than 50% are shown next to the branches. *F. oxysporum* and *F. inflexum* are the outgroup.

Phylogenetic relationships were analyzed by the neighbor-joining method which was based on 670 aligned positions (Figure 1). In the data set, there were 135 variable sites, 526 conserved sites and 111 parsimony informative sites. All the main clades of the tree were well supported with bootstrap values ranging from 98%–100%. The phylogenetic tree can be divided into two main clades, I and II. Clade I consisted of sub-clades A and B. Isolates of *F. sacchari* were grouped in sub-clade A. Isolates of *F. fujikuroi* and *F. proliferatum* were grouped separately in sub-clades B1 and B2 respectively. Isolates of *F. andiyazi* and *F. verticillioides* were grouped separately in sub-clades C1 and C2. *F. oxysporum* and *F. inflexum* were clustered separately from the isolates of *G. fujikuroi* species complex.

The isolates of *F. sacchari* formed a distinct clade that was clearly separated from the other isolates of *Fusarium* in the neighbor-joining tree. The taxa closest to *F. sacchari* were *F. proliferatum* and *F. fujikuroi*. The grouping of *F. sacchari*, *F. proliferatum* and *F. fujikuroi* were in accordance with the biogeographic hyphotesis formulated by O’Donnell *et al.* [14] which grouped the three species in the Asian clade.

Eleven isolates of *F. fujikuroi* and nine isolates of *F. proliferatum* were identified based on the closest match on of TEF-1α gene sequences. Both isolates of *F. fujikuroi* and *F. proliferatum* formed separate sub-clades in the tree (Figure 1) but are in the same main clade which indicated that *F. fujikuroi* and *F. proliferatum* are closely related. Similar results were also reported by Wulff *et al*. [10] in which *F. fujikuroi* and *F. proliferatum,* which both cause bakanae disease, were grouped in separate sub-clades. Both *F. fujikuroi* and *F. proliferatum* are grouped in the Asian clade and are morphologically and biologically very similar [14,24]. Moreover, *F. fujikuroi* and *F. proliferatum* are regarded as sibling species and some isolates can cross fertile [25].

Isolates of *F. verticillioides* were clustered in the same main clade with isolates of *F. andiyazi. F. verticillioides* was grouped in the African clade with several other agricultural important pathogens of *G. fujikuroi* species complex [14]. Although *F. verticillioides* is a member of the African clade, the major host of the fungus is maize which originated in Mexico or Central America. With more species recovered from different host plants and substrates in Malaysia, it could change the composition of the African clade. As for *F. andiyazi*, the detail species concept and phylogenetic analysis of the species remains to be determined [26].

This study presents the identification of *Fusarium* species of *G. fujikuroi* species complex from rice, sugarcane and maize based on TEF-1α gene which is a useful marker for distinguishing *Fusarium* species [12]. Phylogenetic analysis based on the gene clearly grouped the different species into separate groups with high bootstrap values confirming that TEF-1α gene is a good marker for identification of *Fusarium* species. However, additional genes such as other protein coding genes and genes involved in mycotoxin biosynthesis would support the taxonomic affinities of isolates which showed 97–99% sequence similarities as well as to study the pathogen populations. Studies on the evaluation of mycotoxins production are also needed as mycotoxins are commonly produced by *Fusarium* species in the *G. fujikuroi* species complex. Furthermore, there is also the probability that new strains will probably emerge through hybridization, which could lead to the establishment of new plant diseases.

## 3. Experimental Section

### 3.1. Fungal Isolates

Fungal isolates used in this study were obtained from three hosts, namely, rice, sugarcane and maize. *Fusarium* isolates from rice were obtained from rice plants showing bakanae disease symptoms, showing abnormal elongation of the stem. *Fusarium* isolates from sugarcane were isolated from leaves with pokkah boeng symptoms showing leaves crumpled, stunted, twisted and chlorosis near the base. From maize, *Fusarium* isolation was made from rotting of the kernels. The samples were collected from 2008–2009.

Abnormal growth of rice plants and sugarcane leaves were cut to approximately 1 cm^2^ with a sterile scalpel and soaked in 10% sodium hypoclorite (Clorox) for 30 s, transferred into sterile distilled water and soaked for another 30 s. The pieces of the tissues were then dried on sterilized filter paper (Whatman^®^ No 1) and put onto peptone pentachloronitrobenzene agar (PPA) plate. The PPA plates were incubated at 25 °C until mycelia growth was observed. Mycelia from the samples were then subcultured onto potato sucrose agar (PSA). Kernels from maize were directly plated onto PPA plates with sterile forceps and incubated at 25 °C until mycelia growth were observed from the samples. The mycelia were also transferred onto PSA. To obtain pure culture of mycelia grow from the tissues and maize kernels, single spore isolation was carried out using dilution plating [4]. For identification, three different types of media were used, namely Potato Dextrose Agar (PDA), PSA and Carnation Leaf Agar (CLA) as described in The *Fusarium* Laboratory Manual [4]. Microscopic and macroscopic characteristics as described in the manual were used for species identification.

Thirty isolates from the field comprising 19 isolates from maize, three isolates from rice and eight isolates from sugarcane were successfully isolated and identified. Another 48 isolates from the rice, sugarcane and maize were also obtained from stock culture of School of Biological Sciences, Universiti Sains Malaysia culture collection. The samples were collected in 2007–2008. The isolates from the stock culture were also recovered from bakanae disease of rice, pokkah boeng disease of sugarcane and rotting of maize kernels and were identified based on morphological characteristics. In total, 78 isolates of *Fusarium* were used in this study (Table 1).

### 3.2. DNA Extraction

Genomic DNA was extracted from mycelia grown on PSA, incubated for 7 days at 27 °C. Mycelia were ground in a mortar with liquid nitrogen until it became a fine powder. Approximately 0.23 g of the lyophilized mycelia was used for DNA extraction with a DNeasy^®^ Plant Mini Kit (Qiagen, Germany), according to the manufacturer’s instructions. The DNA concentration was measured from all samples using UV spectrophotometry using a Nanodrop ND-1000 (Thermo Scientific, Wilmington, DE, USA) and adjusted to 20 ng/μL.

### 3.3. PCR Amplification

The TEF-1α gene was amplified with primers EF-1 (5′GTT AAG AGG CGC GGT GTC GGT GTG 3′) and EF-2 (5′GGA AGT ACC AGT GAT CAT GTT3′) [14]. Amplification reactions were carried out in a 50 μL reaction mixture which consisted of 1X PCR buffer, 3.5 mM of MgCl_2_, 0.16 mM of dNTP mix (Promega, Madison WI, USA), 1.75 unit of GoTaq^®^ DNA polymerase (Promega, Madison WI, USA), 0.3 μM of each primers and 0.5 μL of DNA template.

PCR was performed in a DNA Engine™ Peltier Thermal Cycler Model PTC-100 (MJ Research) as follows: initial denaturation at 94 °C for 85 s followed by 35 cycles of denaturation at 95 °C for 35 s, annealing at 59 °C for 55 s and extension at 72 °C for 90 s, followed by final extension for 10 min at 72 °C.

PCR products were detected by electrophoresis in a 1.5% agarose gel in TBE buffer. The electrophoresis was run for 100 min at 80 V and 400 mA. The size of the band was estimated by comparison to 100 bp DNA marker (MBI Fermentas, Lithunia). A negative control was used to test the absence of contamination.

### 3.4. DNA Sequencing and Phylogenetic Analysis

The PCR products were purified using a QIAquick PCR Purification Kit (Qiagen, Germany) according to the manufacurer’s instruction. The purified PCR products were directly sequence in both directions using the respective forward and reverse primers, and sent for sequencing to a service provider.

DNA sequences were aligned by using ClusterW Multiple alignment in Molecular Evolutionary Genetic Analysis 4 or MEGA 4 [27] and adjusted manually. Default gap penalties were used in the pair-wise alignment. The consensus sequence was then compared with other DNA sequences by using BLAST against FUSARIUM-ID database at http://fusarium.cbio.psu.edu [12].

All sequences were aligned with gap opening penalty of 3.0, and a gap extension penalty of 1.8. Neighbor-joining analysis was then performed in MEGA 4. A neighbor-joining tree was constructed with the Jukes-Cantor model and the reliability of neighbor-joining trees was estimated by bootstrap method with 1000 replications. *Fusarium oxysporum* (FJ985415) and *F. inflexum* (AF331814) sequences from GenBank were selected as the outgroup.

## 4. Conclusions

The wide host range of *Fusarium* species in the *G. fujikuroi* species complex suggest that new species or species that have not been reported from Peninsular Malaysia are likely to be found on agricultural crops and other substrates such as from the soils. The present study suggests that many isolates of *Fusarium* species in the *G. fujikuroi* species complex in culture collections maybe misidentified which is not surprising since the isolates have been identified primarily using morphological characters. Accurate identification of *Fusarium* species associated with economically important crops could help in the formulation of disease control strategies, prediction of host range, climatic adaptation and mycotoxin production potential.

## Figures and Tables

**Table 1:** Identification of *Fusarium* isolates from rice, sugarcane and maize based on morphological and (translation elongation factor) TEF-1α sequences.

Isolate and Host	Location	Morphological Identification	TEF-1α sequences (% similarity)	Accession Number
*0654 (R)	Seberang Perai, Penang	*F. verticillioides*	*F. verticillioides* (99)	JN675681
*3063 (R)	Seberang Perak, Perak	*F. verticillioides*	*F. verticillioides* (99)	JN675682
*3068 (R)	Jabi, Terengganu	*F. verticillioides*	*F. verticillioides* (99)	JN675683
*3124 (R)	Kuala Selangor, Selangor	*F. verticillioides*	*F. verticillioides* (99)	JN675680
*3257 (SC)	Padang Terap, Kedah	*F. verticillioides*	*F. verticillioides* (100)	JN675684
T1 (SC)	Chuping, Perlis	*F. verticillioides*	*F. verticillioides* (100)	JN675701
T2 (SC)	Chuping, Perlis	*F. verticillioides*	*F. verticillioides* (99)	JN675702
*3277 (SC)	Chuping, Perlis	*F. verticillioides*	*F. verticillioides* (100)	JN675699
T5 (SC)	Chuping, Perlis	*F. verticillioides*	*F. verticillioides* (100)	JN675700
JB1 (M)	Gurun, Kedah	*F. verticillioides*	*F. verticillioides* (99)	JN675693
JB2 (M	Gurun, Kedah	*F. verticillioides*	*F. verticillioides* (99)	JN675694
JB3 (M)	Gurun, Kedah	*F. verticillioides*	*F. verticillioides* (99)	JN675695
JB4 (M)	Gurun, Kedah	*F. verticillioides*	*F. verticillioides* (99)	JN675696
JD4 (M)	Gurun, Kedah	*F. verticillioides*	*F. verticillioides* (99)	JN675697
JF1 (M)	Gurun, Kedah	*F. verticillioides*	*F. verticillioides* (99)	JN675698
B1 (M)	Gurun, Kedah	*F. verticillioides*	*F. verticillioides* (99)	JN675685
B2 (M)	Gurun, Kedah	*F. verticillioides*	*F. verticillioides* (100)	JN675686
B3 (M)	Gurun, Kedah	*F. verticillioides*	*F. verticillioides* (98)	JN675687
B4 (M)	Gurun, Kedah	*F. verticillioides*	*F. verticillioides* (100)	JN675688
B5 (M)	Gurun, Kedah	*F. verticillioides*	*F. verticillioides* (100)	JN675689
B6 (M)	Gurun, Kedah	*F. verticillioides*	*F. verticillioides* (100)	JN675690
B9 (M)	Gurun, Kedah	*F. verticillioides*	*F. verticillioides* (99)	JN675691
F1 (M)	Gurun, Kedah	*F. verticillioides*	*F. verticillioides* (99)	JN675692
*3055 (R)	Seberang Perak, Perak	*F. verticillioides*	*F. andiyazi* (98	JN408195
*3061 (R)	Seberang Perak, Perak	*F. verticillioides*	*F. andiyazi* (98)	JN408196
*3073 (R)	Tumpat, Kelantan	*F. verticillioides*	*F. andiyazi* (98)	JN675629
*3086 (R)	Rompin, Pahang	*F. verticillioides*	*F. andiyazi* (98)	JN408197
*3088 (R)	Rompin, Pahang	*F. verticillioides*	*F. andiyazi* (98)	JN408198
*3137 (R)	Sungai Besar, Selangor	*F. verticillioides*	*F. andiyazi* (98)	JN675630
*3053 (R)	Teluk Intan, Perak	*F. sacchari*	*F. sacchari* (99)	JN675640
*3054(R)	Seberang Perak, Perak	*F. sacchari*	*F. sacchari* (99)	JN675641
*3078 (R)	Rompin, Pahang	*F. sacchari*	*F. sacchari* (100)	JN675642
*3081 (R)	Rompin, Pahang	*F. sacchari*	*F. sacchari* (99)	JN675643
*3082 (R)	Rompin, Pahang	*F. sacchari*	*F. sacchari* (99)	JN675644
*3084 (R)	Rompin, Pahang	*F. sacchari*	*F. sacchari* (99)	JN675645
*3087 (R)	Rompin, Pahang	*F. sacchari*	*F. sacchari* (99)	JN675646
*3136 (R)	Sungai Besar, Selangor	*F. sacchari*	*F. sacchari* (97)	JN675647
P5 (R)	Gurun, Kedah	*F. sacchari*	*F. sacchari* (99)	JN675662
*3246 (SC)	Padang Terap, Kedah	*F. sacchari*	*F. sacchari* (99)	JN675648
*3262 (SC)	Padang Terap, Kedah	*F. sacchari*	*F. sacchari* (99)	JN675649
*3281 (SC)	Chuping, Perlis	*F. subglutinans*	*F. sacchari* (99)	JN675650
*3295 (SC)	Chuping, Perlis	*F. subglutinans*	*F. sacchari* (99)	JN675651
*3308 (SC)	Kupang, Kedah	*F. subglutinans*	*F. sacchari* (99)	JN675652
*3310 (SC)	Kupang, Kedah	*F. subglutinans*	*F. sacchari* (99)	JN675653
*3327 (SC)	Rantau Panjang, Kelantan	*F. sacchari*	*F. sacchari* (99)	JN675654
*3336 (SC)	Kuantan, Pahang	*F. sacchari*	*F. sacchari* (98)	JN675655
*3343 (SC)	Kg Awah, Pahang	*F. sacchari*	*F. sacchari* (99)	JN675656
*3349 (SC)	Kuantan, Pahang	*F. subglutinans*	*F. sacchari* (100)	JN675657
*3350 (SC)	Kuantan, Pahang	*F. sacchari*	*F. sacchari* (100)	JN675658
T3 (SC)	Chuping, Perlis	*F. sacchari*	*F. sacchari* (99)	JN675663
T4 (SC))	Chuping, Perlis	*F. sacchari*	*F. sacchari* (99)	JN675664
T6 (SC)	Chuping, Perlis	*F. sacchari*	*F. sacchari* (99)	JN675665
T7 (SC)	Chuping, Perlis	*F. sacchari*	*F. sacchari* (99)	JN675666
T8 (SC)	Chuping, Perlis	*F. sacchari*	*F. sacchari* (99)	JN675667
T9 (SC))	Chuping, Perlis	*F. sacchari*	*F. sacchari* (99)	JN675668
*3358 (SC)	Bagan Serai, Johor	*F. sacchari*	*F. sacchari* (99)	JN675659
B10 (M)	Gurun, Kedah	*F. subglutinans*	*F. sacchari* (99)	JN675660
F2 (M)	Gurun, Kedah	*F. subglutinans*	*F. sacchari* (100)	JN675661
*0621 (R)	Cuping, Perlis	*F. fujikuroi*	*F. fujikuroi* (98)	JN675669
*3067 (R)	Jabi, Terengganu	*F. fujikuroi*	*F. fujikuroi* (98)	JN675670
*3099 (R)	Kuala Selangor, Selangor	*F. fujikuroi*	*F. fujikuroi* (98)	JN675671
*3101 (R)	Kuala Selangor, Selangor	*F. fujikuroi*	*F. fujikuroi* (98)	JN675672
*3105 (R)	Kuala Selangor, Selangor	*F. fujikuroi*	*F. fujikuroi* (98)	JN675673
*3122 (R)	Kuala Selangor, Selangor	*F. fujikuroi*	*F. fujikuroi* (97)	JN675674
*3132 (R)	Sungai Besar, Selangor	*F. fujikuroi*	*F. fujikuroi* (98)	JN675678
*3151 (R)	Sungai Besar, Selangor	*F. proliferatum*	*F. fujikuroi* (98)	JN675675
*3170 (R)	Permatang Tok Jaya, Penang	*F. proliferatum*	*F. fujikuroi* (98)	JN675676
*3208 (R)	Permatang Tok Jaya, Penang	*F. fujikuroi*	*F. fujikuroi* (98)	JN675677
JF5 (M)	Gurun, Kedah	*F. proliferatum*	*F. fujikuroi* (98)	JN675679
*3074 (R)	Tumpat, Kelantan	*F. proliferatum*	*F. proliferatum* (99)	JN675636
*3075 (R)	Tumpat, Kelantan	*F. proliferatum*	*F. proliferatum* (98)	JN675637
*3095 (R)	Kuala Selangor, Selangor	*F. proliferatum*	*F. proliferatum* (98)	JN675631
P1 (R)	Gurun, Kedah	*F. proliferatum*	*F. proliferatum* (98)	JN675633
P2 (R)	Gurun, Kedah	*F. proliferatum*	*F. proliferatum* (98)	JN675634
P4 (R)	Gurun, Kedah	*F. proliferatum*	*F. proliferatum* (99)	JN675635
*3238 (SC)	Padang Terap, Kedah	*F. proliferatum*	*F. proliferatum* (98)	JN675638
*3324 (SC)	Rantau Panjang, Kelantan	*F. proliferatum*	*F. proliferatum* (98)	JN675639
*3354 (SC)	Bagan Serai, Perak	*F. proliferatum*	*F. proliferatum* (99)	JN675632

R: rice; SC: sugarcane; M: maize;

* isolates from stock culture.

## References

[1] Marasas WFO, Ploetz RC, Wingfield MJ, Wingfield BD, Steenkamp ET (2006). Mango malformation disease and the associated *Fusarium* species. Phytopathology.

[2] Mohd Zainudin NAI, Razak AA, Salleh B (2008). Bakanae disease of rice in Malaysia and Indonesia: Etiology of the causal agent based on morphological, physiological and pathogenicity characteristics. J. Plant Prot. Res.

[3] Siti Nordahliawate MS, Nur Ain Izzati MZ, Razak AA, Salleh B (2008). Distribution, morphological characterization and pathogenicity of *Fusarium sacchari a*ssociated with pokkah boeng disease of sugarcane in Peninsular Malaysia. J. Trop. Agric. Sci.

[4] Leslie JF, Summerell BA (2006). The Fusarium Laboratory Manual.

[5] Yli-Matilla T, Paavaeen-Huhtala S, Bulat SA, Alekhina IA, Nirenberg HI (2002). Molecular, morphological and phylogenetic analysis of the *Fusarium avenaceum* / *F. arthrosporioides* / *F. tricinctum* species complex – a polyphasic approach. Mycol. Res.

[6] Knutsen AK, Torp M, Holst-Jensen A (2004). Phylogenetic analyses of *Fusarium poae, Fusarium sporotrichiodes* and *Fusarium langsethiae* species complex based on partial sequences of the translation elongation factor 1-α gene. Int. J. Food Microbiol.

[7] Jurjevic Z, Wilson DM, Wilson JP, Geiser DM, Juba JH, Mubatanhema W, Widstrom NW, Rains GC (2005). *Fusarium* species of the *Gibberella fujikuroi* complex and fumonisin contamination of pearl millet and corn in Georgia, USA. Mycopathologia.

[8] Morales-Rodriguez I, deYanex-Morales M, Silva-Rajas HV, Garcia de-la-Santos G, Guzman de-Pena DA (2007). Biodiversity of *Fusarium* species in Mexico associated with ear rot in maize and their identification using phylogenetic approach. Mycopathologia.

[9] Nalim FA, Elmer WH, McGovern PJ, Geiser DM (2009). Multilocus phylogenetic diversity of *Fusarium avenaceum* pathogenic on lisianthus. Phytopathology.

[10] Wulff EG, Sørensen JL, Lübeck M, Nielsen KF, Thrane U, Torp J (2010). *Fusarium* spp. associated with rice Bakanae: Ecology, genetic diversity, pathogenicity and toxigenicity. Environ. Microbiol.

[11] Harrow SA, Ferrokhi-Nejad R, Pitman AR, Scott IAW, Bentley A, Hide C, Cromey MG (2010). Characterization of New Zealand *Fusarium* populations using a polyphasic approach differentiates the *F. avenaceum/F. acuminatum/F. tricinctum* species complex in cereal and grassland systems. Fungal Biol.

[12] Geiser DM, Jimenez-Gasco M, Kang S, Makalowska I, Veeraraghavan N, Ward TJ, Zhang N, Kuldau GA, O’Donnell K (2004). FUSARIUM-ID v. 1.0: A DNA sequence database for identifying *Fusarium*. Eur. J. Plant Pathol.

[13] Rossman AY, Palm-Hernandez ME (2008). Systematics of plant pathogenic Fungi. Why it matters. Plant Dis.

[14] O’Donnell K, Cigelnik E, Nirenberg HI (1998). Molecular systematics and phylogeography of the *Gibberella fujikuroi* species complex. Mycologia.

[15] O’Donnell K, Nirenberg HI, Aoki T, Cigelnik E (2000). A multigene phylogeny of the *Gibberella fujikuroi* species complex: Detection of additional phylogenetically distinct species. Mycoscience.

[16] Marasas WFO, Rheeder JP, Lamprecht SC, Zeller KA, Leslie JF (2001). *Fusarium andiyazi sp. nov*., a new species from sorghum. Mycologia.

[17] Steenkamp ET, Wingfield BD, Coutinho TA, Wingfield MJ, Marasas WFO (1999). Differentiation of *Fusarium subglutinans* f. sp. *pini* by histone gene sequence data. Appl. Environ. Microb.

[18] Klittich CJ, Leslie JF, Nelson PE, Marasas WFO (1997). *Fusarium thapsinum* (*Gibberella thapsina*): a new species in section Liseola from sorghum. Mycologia.

[19] Bacon CW, Hinton DM (1996). Symptomless endophytic colonization of maize by *Fusarium moniliforme*. Can. J. Botany.

[20] Latiffah Z, Mah KF, Heng MH, Maziah Z, Baharuddin S (2010). *Fusarium* species isolated from mangrove soil in Kampung Pantai Acheh, Balik Pulau, Pulau Pinang. Trop. Life Sci. Res.

[21] Burgess LW, Summerell BA, Bullock S, Gott KP, Backhouse D (1994). Laboratory Manual for Fusarium Research.

[22] Logrieco A, Mule G, Moretti A, Bottalico A (2002). Toxigenic *Fusarium* species and mycotoxins associated with maize ear rot in Europe. Eur. J. Plant Pathol.

[23] Leslie JF (1991). Mating populations in *Gibberella fujikuroi* (*Fusarium* section Liseola). Phytopathology.

[24] Leslie JF, Anderson LL, Bowden RL, Lee YW (2007). Inter- and intra-specific genetic variation in *Fusarium*. Int. J. Food Mircobiol.

[25] Leslie JF, Zeller KA, Wahler M, Summerell BA (2004). Interfertility of two mating populations in the *Gibberella fujikuroi* species complex. Eur. J. Plant Pathol.

[26] Kvas M, Marasas WFO, Wingfield BD, Wingfield MJ (2009). Diversity and evolution of *Fusarium* species in the *Gibberella fujikuroi* complex. Fungal Divers.

[27] Tamura K, Dudley J, Nei M, Kumar S (2007). MEGA 4: Molecular Evolutionary Genetics Analysis (MEGA) software 4.0. Mol. Biol. Evol.

